# Food Protein-Induced Enterocolitis Syndrome in Children: A Case Report

**DOI:** 10.7759/cureus.113819

**Published:** 2026-08-02

**Authors:** Karima El Fakiri, Zineb Bouhsini, Imane Fetoui, Aicha Abourrahouat, Mohammed Bouskraoui

**Affiliations:** 1 Department of Pediatrics A, Mohammed VI University Hospital, Faculty of Medicine and Pharmacy, Cadi Ayyad University, Marrakech, MAR; 2 Department of Pediatrics B, Mohammed VI University Hospital, Faculty of Medicine and Pharmacy, Cadi Ayyad University, Marrakech, MAR

**Keywords:** child, dietary management, elimination diet, food protein-induced enterocolitis syndrome, oral food challenge

## Abstract

Food protein-induced enterocolitis syndrome (FPIES) is a rare form of non-IgE-mediated food allergy that remains underrecognized and is often responsible for diagnostic delay. We report the case of an eight-year-old child with a history of celiac disease, presenting with recurrent episodes of abdominal pain and vomiting occurring in a delayed manner after ingestion of specific foods.

The clinical presentation was characterized by delayed, intermittent, and reproducible gastrointestinal symptoms. Standard allergy investigations, including specific IgE testing and prick-to-prick testing with the suspected food allergens, were negative, supporting a non-IgE-mediated mechanism strongly suggestive of FPIES.

Clinical evolution was marked by improvement following elimination of the implicated foods. This case highlights the diagnostic challenges of this condition and emphasizes the importance of a rigorous clinical approach in children presenting with unexplained gastrointestinal symptoms.

## Introduction

Food protein-induced enterocolitis syndrome (FPIES) is a distinct form of non-IgE-mediated food allergy involving predominantly cell-mediated immune mechanisms [1,2]. Unlike IgE-mediated food allergies, FPIES typically presents with delayed symptoms occurring one to four hours after ingestion of the triggering food, mainly characterized by repetitive vomiting, lethargy, and, in severe cases, dehydration or hypovolemic shock [2,3]. The absence of specific biomarkers and the negativity of conventional allergy tests make the diagnosis essentially clinical, contributing to frequent diagnostic delays [1-3]. The most commonly implicated foods include cow’s milk and soy, although other foods may be involved, particularly in older children [2,3]. The natural course is generally favorable, with gradual acquisition of tolerance during childhood, although persistent or atypical forms have been described [1-3]. We report a case illustrating an atypical presentation of FPIES in a school-aged child with a history of celiac disease.

## Case presentation

We report the case of an eight-year-old child who had been followed for celiac disease for the past five years and was on a strict gluten-free diet.

The clinical history was marked, over the past four months, by recurrent episodes of abdominal pain associated with vomiting. These episodes occurred intermittently, in flare-ups separated by remission periods, with a total of four reactions. Symptoms appeared in a delayed manner, several hours after ingestion of specific foods, particularly milk, eggs, peanuts, walnuts, lentils, beans, and fava beans, without any associated cutaneous or respiratory manifestations. No features suggestive of immediate anaphylaxis were observed. On physical examination, the child was in good general condition, with preserved growth parameters. Abdominal examination was unremarkable outside symptomatic episodes. Symptomatic treatment was administered during acute episodes. The allergological work-up, including food-specific IgE testing and prick-to-prick tests with common suspected foods, was negative, ruling out IgE-mediated food allergy. Additional laboratory investigations were within normal limits and showed no evidence of inflammatory or infectious processes, nor relapse of celiac disease (Table 1). After excluding infectious gastroenteritis (including Salmonella and Shigella), IgE-mediated food allergy, and eosinophilic esophagitis, the diagnosis of FPIES was considered. Given the delayed onset of gastrointestinal symptoms, their reproducibility after ingestion of specific foods, the absence of IgE sensitization, and the improvement observed with dietary avoidance, the diagnosis of acute FPIES was retained. This diagnosis was supported by one major criterion, vomiting occurring 1 to 4 hours after ingestion of the suspected food, and three minor criteria, namely, marked lethargy, diarrhea within 24 hours, and pallor during episodes. Initial management consisted of strict avoidance of the suspected triggering foods, along with antiemetic therapy and oral rehydration during acute episodes. A structured and gradual reintroduction strategy was subsequently implemented. An oral food challenge (OFC) with cow’s milk was first performed, given its frequent implication in FPIES, followed one week later by a legume challenge, and later by an egg OFC after 12 months of avoidance. The protocol included admission to a day-hospital setting, placement of a peripheral venous line due to the risk of hypovolemia, and administration of the test food in three incremental doses at 30-minute intervals. Prolonged monitoring was ensured for at least four hours after the last dose, with the possibility of extending observation up to 24 hours due to the delayed nature of FPIES reactions. No immediate or delayed adverse reactions were observed during these challenges.

**Table 1:** Biological and allergological investigations in the reported case

Parameter	Patient Value	Normal Range (Child ~8 years)	Interpretation
Hemoglobin	11.8 g/dL	11.5 – 15 g/dL	Normal (lower limit)
White blood cells (WBC)	6,230 /mm³	4,500 – 13,500 /mm³	Normal
Neutrophils	2,947 /mm³	1,500 – 8,000 /mm³	Normal
Platelets	249,000 /mm³	150,000 – 450,000 /mm³	Normal
Serum IgA	Normal	Age-dependent (≈ 0.7 – 4 g/L)	Normal
Anti-transglutaminase IgA	Negative	Negative	No evidence of active celiac disease
Food-specific IgE	Negative	Negative	No IgE-mediated allergy
Prick-to-prick tests	Negative	Negative	No sensitization

Follow-up included patient and family education regarding dietary management, food labeling, and recognition of symptoms. An individualized emergency action plan was provided, including oral rehydration measures and referral to the emergency department in case of severe reactions requiring intravenous fluid resuscitation and ondansetron administration.

## Discussion

FPIES is a non-IgE-mediated food allergy involving cell-mediated immune responses, leading to gastrointestinal inflammation [1,2]. This immune response is thought to involve T-cell activation and cytokine-mediated inflammation, particularly TNF-α, resulting in increased intestinal permeability and antigen passage across the epithelial barrier [3,4]. Its clinical presentation is characterized by delayed symptoms, mainly digestive, which contributes to its underrecognition [1,5]. These manifestations typically occur one to four hours after ingestion of the offending food and are frequently misdiagnosed as infectious or surgical conditions, contributing to diagnostic delay [5,6]. Although typically occurring in infancy, late-onset cases in older children have been reported, suggesting the existence of atypical or persistent forms [1,2]. While most cases occur during the first months of life, especially at the introduction of cow’s milk or solid foods, cases described beyond infancy highlight the heterogeneity of the disease and possible delayed recognition [6,7]. The diagnostic delay observed in our case is consistent with the literature, often due to the absence of specific biomarkers and the non-specific nature of symptoms [1,5]. In addition, the lack of a diagnostic gold standard and the reliance on clinical criteria contribute to frequent misdiagnosis and prolonged diagnostic wandering [6,8]. The typical clinical pattern includes repetitive vomiting occurring hours after ingestion, without cutaneous or respiratory involvement. Severe cases may lead to hypovolemic shock [1,5]. Additional features such as lethargy, hypotonia, dehydration, and metabolic disturbances may also be observed in severe presentations [5,6,9]. Negative skin tests are a hallmark feature and help differentiate FPIES from IgE-mediated allergy [1,5]. However, atypical forms with IgE sensitization have been described and are associated with a more prolonged disease course and delayed acquisition of tolerance [7,8]. Management relies on strict elimination of the triggering foods, with symptomatic treatment during acute episodes, including hydration and, when necessary, antiemetic therapy [1]. In severe cases, intravenous fluid resuscitation is essential, and ondansetron is effective in controlling persistent vomiting [5,6,9]. Oral food challenges remain the gold standard for diagnosis confirmation and reintroduction of foods, particularly in atypical cases [1]. These procedures should be conducted in a controlled medical setting due to the risk of delayed but potentially severe reactions [5]. Tolerance is generally assessed after 12-18 months of avoidance [1]. However, the timing of reintroduction may vary depending on the type of food and clinical severity [3]. Importantly, some patients may develop IgE sensitization over time, requiring long-term follow-up [1,2]. This evolution toward IgE-mediated allergy has been described in a subset of patients, particularly in atypical forms, emphasizing the need for continuous allergological monitoring [7].

## Conclusions

FPIES is a rare and often underrecognized form of non-IgE-mediated food allergy, whose diagnosis relies primarily on careful clinical history. Our case illustrates an atypical presentation occurring in a school-aged child, characterized by delayed gastrointestinal manifestations, negative standard allergy testing, and favorable evolution with dietary elimination. This observation highlights the clinical variability of FPIES and the possibility of persistent or late-onset forms, which may complicate diagnosis. Early recognition of this condition helps avoid unnecessary investigations and allows appropriate management based on targeted elimination and structured food reintroduction. FPIES may evolve toward an IgE-mediated sensitization over time, warranting long-term allergological follow-up.
